# Premature Ejaculation: Aetiology and Treatment Strategies

**DOI:** 10.3390/medsci7110102

**Published:** 2019-10-25

**Authors:** Nicholas Gillman, Michael Gillman

**Affiliations:** 1 Griffith University School of Medicine, Gold Coast QLD 4215, Australia; nicholas.gillman@griffithuni.edu.au; 2 St Andrew’s War Memorial Hospital, Spring Hill QLD 4001, Australia

**Keywords:** premature ejaculation, aetiology, treatment

## Abstract

Premature ejaculation (PE) is a highly prevalent male sexual dysfunction that is often neglected, presenting a currently unmet therapeutic need. The classification of PE has historically been varied and at times ambiguous, contributing to inaccurate prevalence estimates. This review uses the International Society for Sexual Medicine (ISSM) definition of PE, which includes reduced ejaculatory latency, lack of control and associated negative personal consequences. Patient assessment and management options differ depending on the classification of PE and it is the role of the clinician to appropriately classify patients and be aware of the correct management strategies. This review provides an overall background of PE in terms of classification and underlying physiology, patient assessment and management strategies along with the scientific rationale for treatment. Patients with lifelong and acquired PE are most likely to benefit from combination therapy of pharmacological treatment in the form of selective serotonin re-uptake inhibitor dapoxetine, psychosexual behavioural therapy and psychological therapy.

## 1. Introduction

Premature ejaculation (PE) is a highly prevalent male sexual dysfunction. Although the true prevalence of the dysfunction is unclear, it may affect as high as 20–30% of men regardless of age and ethnicity [1,2]. PE can impact multiple facets of a patient’s life, including psychological and emotional health, as well as interpersonal relationships with partners and/or spouses. Despite this, it is an area of male sexual health which is generally neglected, presenting an unmet therapeutic need. This is likely due to a combination of many factors, including poor rates of seeking treatment as a result of embarrassment or shame, as well as confusion on the part of the clinician surrounding clinical management of the dysfunction.

## 2. International Society of Sexual Medicine Definition of PE and Classification

There has historically been distinct variation in epidemiological study outcomes of PE around the globe [3,4]. The lack of clarity can be partly attributed to variation and ambiguity in the definition of PE [4,5,6,7]. As a result, an Ad Hoc Committee for the Definition of Premature Ejaculation was formed by the International Society for Sexual Medicine (ISSM) in 2007. Their role was to provide an evidence-based, unified definition of PE. Three key criteria were agreed upon and established, including intravaginal ejaculation latency time (IELT), lack of ejaculatory control and negative personal consequences associated with PE. A unified definition was published in 2013 by the Committee to define PE, amending the previous definition. The Committee defined PE as a male sexual dysfunction characterised by:

1. Ejaculation which always or nearly always occurs prior to or within about one minute of vaginal penetration from the first sexual experience (lifelong), or, a clinically significant reduction in latency time, often to about 3 min or less (acquired).
2. The inability to delay ejaculation on all or nearly all vaginal penetrations.
3. Negative personal consequences, such as distress, bother, frustration and/or the avoidance of sexual intimacy [4].

The definition classifies patients into two categories to include patients in which ejaculation with reduced latency has occurred from the first sexual experience (lifelong PE) and patients reporting a clinically significant reduction from previous latency (acquired PE). This distinction is made as PE is not homogenous across populations. Although not captured by the ISSM definition, PE can be further classified into four distinct categories, described by Waldinger and Schweitzer as lifelong (primary), acquired (secondary), variable and subjective [8,9].

Lifelong PE is characterised by low IELT, usually less than one minute, from the first sexual experience, and on most, if not all, subsequent encounters [10]. This form of PE is suggestive of a functional disturbance, and may be linked with disturbances in timing of erection, detumescence and arousal [6]. Patients with lifelong PE may present with several sexual dysfunctions including erectio praecox (early erections), ejaculatio praecox (early ejaculations), detumescentia praecox (early detumescence) and a hypererotic state [6]. Lifelong PE has also been linked to familial occurrence, as well as possible genetic polymorphisms [11,12].

Acquired PE however, refers to reduced ejaculatory latency that develops at some point in the patient’s life. Patients with acquired PE have experienced normal ejaculations in the past, and the dysfunction is usually due to an identifiable medical, psychological or interpersonal underlying cause. Risk factors for acquired PE include psycho-relational, endocrine and urologic dysfunction [13]. Patients may also present with other sexual comorbidities, most commonly erectile dysfunction. Other causes of acquired premature ejaculation have occasionally been reported, including prostatitis and hyperthyroidism [13].

As outlined earlier, variable and subjective PE have been recently added to the classification of PE. It is important to be reminded that the ISSM definition of PE does not extend to variable and subjective PE. Variable PE describes early ejaculation that occurs inconsistently, or irregularly in a man’s sexual life. This form of PE is not thought of as a disorder, but rather a normal variation in ejaculatory time. Subjective PE differs in that patients present with self-described/self-perceived early ejaculation despite normal IELT. The diagnostic picture and management of PE differs depending on the category of PE.

## 3. Pathophysiology of Premature Ejaculation

### 3.1. Sexual Response Cycle

**Figure 1:**
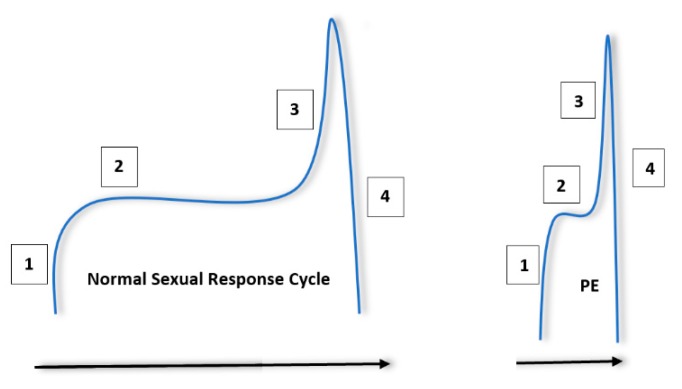
Normal sexual response cycle in men compared to men with premature ejaculation (PE). The steps are labelled in the order in which they occur. (1) Sexual arousal/excitement and penile tumescence. (2) Plateau period. (3) Increase in excitement/arousal to the point or ejaculation and orgasm. (4) Postejaculatory detumescence and resolution. It is important to note that patients with PE will experience a sharp excitement phase, followed by a short plateau and subsequent ejaculation.

The normal male sexual response can be thought of in terms of a four step, sequential process [14]. This process begins with excitement, in which penile tumescence and subsequent erection occurs after sexual interest and/or stimulation. A plateau period follows in which ejaculation is delayed and where sexual intercourse may occur. Ejaculation and orgasm occur after this plateau, followed by resolution and associated postejaculatory detumescence. It is thought that this process becomes compressed in patients with PE [15]. Patients may encounter a steep excitement period, followed by reduced latency during the plateau phase and rapid ejaculation. This is summarised in Figure 1.

### 3.2. Process of Ejaculation

In order to appreciate the scientific rationale for PE treatment, the process as well as control of ejaculation must be understood. Ejaculation is a spinal reflex subject to heavy cerebral modulation. It is comprised of two consecutive phases; emission and expulsion. The coordination of this process ensures antegrade propulsion of semen. During emission, spermatozoa and seminal fluid are secreted into the prostatic urethra [16]. Expulsion follows, a process in which rhythmic contractions of bulbospongiosus and ischiocavernosus muscles as well as pelvic striated muscles lead to the movement of semen through the urethra and out the urethral meatus [17].

### 3.3. Peripheral Control

This process is thought to mainly be stimulated by autonomic sympathetic and parasympathetic efferent fibres from the pelvic plexus [18,19]. It is thought that the sympathetic effect is dominant during ejaculation, responsible for controlling the contractile activity of seminal tract smooth muscle [20]. Functional studies have indicated the importance of sympathetic innervation in stimulating contraction of sex glands [16]. The role of the parasympathetic nervous system on ejaculation remains somewhat unclear, although it may play a role in preventing reflux of seminal fluid through the ejaculatory duct, as well as the secretion of seminal fluid [19,20]. Finally, somatic fibres carried by the pudendal nerve are thought to play a role in ejaculation through control of pelvic striated muscles [19].

### 3.4. Spinal Control

The process of ejaculation is a highly coordinated process. This coordination is achieved and modulated at key spinal centres, which together form the spinal network of ejaculation. Sympathetic outflow is achieved from centres in the dorsal grey column and the intermediolateral column of thoracolumbar segments [16]. Parasympathetic outflow occurs at the sacral level from sacral parasympathetic nuclei, while somatic fibres innervating the urethral sphincter and pelviperineal striated muscles are located in Onuf’s nucleus, located in the ventral horn of S2–S4 segments. Recent research has identified a group of lumbar spinothalamic neurons in rats which may lead synchronisation of these spinal centres [21,22,23]. This group of neurons is known as the spinal generator of ejaculation (SGE). There is evidence to suggest that humans may also contain an SGE, which may provide a new target for therapeutic treatment of ejaculatory disorders [24].

### 3.5. Cerebral Control

**Figure 2:**
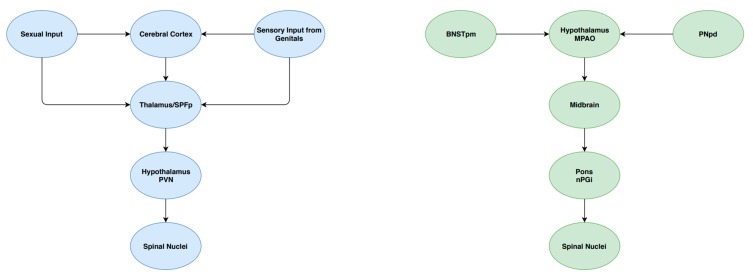
A schematic summary of some components of the central control of ejaculation.

Cerebral control of ejaculation is achieved by a complex ejaculatory network, comprising several groups of interconnected neurons found in various levels of the brain. These supraspinal centres play different roles in the ejaculatory process, including sensory/integrative, excitatory and inhibitory actions [16]. Sensory/integrative control is primarily mediated by the posteromedial part of the bed of the stria terminalis (BNSTpm), the posterodorsal area of the medial amygdala (MPOA), the parvicellular part of the subparafascicular thalamus (SPFp) and the posterodorsal pre-optic nucleus (PNpd) [16]. These areas are responsible for the processing of sexual stimuli. Excitatory pathways in the brain have been identified as playing a critical role in ejaculation. An example of these pathways includes neurons extending from the medial pre-optic area into the paraventricular hypothalamic nucleus [16]. Projections are then sent to autonomic neurons contained in the spinal centres of ejaculation. Finally, nuclei of the ventral medulla have been identified as a source of inhibitory control of ejaculation [25]. In particular, the gigantocellular nuclei and ventral raphé nuclei contain descending axonal projections to both lumbar spinothalamic neurons and autonomic spinal ejaculatory centres. These modulatory centres, as well as their interaction spinal nuclei, are summarised in Figure 2.

## 4. Patient Assessment

Patients with PE may be uncomfortable discussing their ejaculatory disorder and as such may avoid seeking treatment. It is therefore important that clinicians establish sexual health as a routine topic during consultations. One common approach of initiating the conversation about sexual health, and to determine intervention levels for patients, is the PLISSIT Model, first introduced by Annon [26]. This model is a stage-wise approach, and individual patient levels should be identified by the clinician.

1. Permission: This is the first stage in which the clinician gives the client permission to feel comfortable about and discuss a particular facet of their sexual health. It is imperative the clinician maintains a professional, non-judgmental attitude during this time in order to facilitate the conversation.
2. Limited Information: In this stage, the patient is provided with limited information about a topic or issue. It is important in this stage that the clinician learns about specific topics the patient wishes to discuss surrounding their sexual health and provide information for the specific subject.
3. Specific Suggestion: Here, the clinician provides patients with specific advice on particular topics relating to the patient’s sexual health. This is completed after full patient evaluation surrounding the topic. Information includes suggested behavioural changes as well as treatment options for a specific concern.
4. Intensive Therapy: This is the last stage in which the patient begins psychological and/or pharmacological therapy to remediate a specific issue the patient is suffering from regarding their sexual health. This may include referral to a specialist for further treatment and management.

As previously discussed, confirming a diagnosis of PE requires the presence of all three components. The first relates to ejaculatory latency, with patients having an IELT of one minute or less on all or nearly all sexual encounters, or a reduction in IELT to about three minutes. The second is a lack of control, or inability to delay ejaculation. Finally, there must be negative personal consequences for the patient, which may include psychological or interpersonal issues.

As such, clinicians must primarily explore the patient’s sexual history; however a full medical history, psychological history and a physical exam is often necessary to provide context surrounding the PE and to consider possible underlying causes of acquired PE. The sexual history should involve an estimate of IELT, as well as the onset and duration of PE. Previous sexual functioning and history of sexual relations are also necessary in order to delineate acquired and lifelong PE. Degree of ejaculatory control as well as degree of patient and partner distress should also be ascertained in order to fulfil a diagnosis of PE. A psychological history is also important in order to assess the likelihood of a psychological cause or association with the PE. This includes identifying patient stressors, as well as assessing a history/presence of anxiety or depression. A focused physical examination can point towards an underlying cause of PE and this may include a genitourinary examination and neurological assessment of the lower limbs.

A common diagnostic tool used in the assessment of premature ejaculation is the premature ejaculation diagnostic tool (PEDT) developed by Symonds et al. [27]. The tool asks questions regarding the ISSM criteria of premature ejaculation to cover all three components of the definition. It is important to note however that the tool fails to distinguish between acquired and lifelong PE, and as such, involvement by the clinician is important in the identification of the correct PE subtype.

It is also crucial to manage patient expectations when assessing patients with PE. Waldinger et al. found IELT to vary in a population of men with partners >6 months from 0.55–44.1 min, with a median latency time of 5.4 min [28]. Some patients and/or partners may have preconceived perceptions about IELT that are well in excess of this median latency time and may consequently have unrealistic expectations of what can be achieved with therapy.

By gaining an understanding of patient expectations concerning therapeutic outcomes, initiating suitable treatment, or modifying current treatment, can be appropriately managed. This is best achieved by setting achievable treatment goals with the patient. Examples of treatment goals include increasing IELT, increasing control over ejaculation, increasing satisfaction with sexual intercourse and decreasing personal distress related to PE.

## 5. Patient Management Options and Scientific Rationale

### 5.1. Role of Serotonin (5-HT) in Ejaculatory Control

Serotonin acts as the primary neurotransmitter involved in cerebral control of ejaculation. Serotonin cell bodies are arranged in clusters in the brainstem and send projections to other neural sites, as well as descending fibres to the spinal cord. Current literature indicates that serotonergic neurons act on post-synaptic neuronal receptors where they primarily exert an inhibitory effect on ejaculation [29,30]. Several serotonin receptor subtypes have been identified to play a role in this modulation of ejaculation in rats, including 5-HT_1A_, 5-HT_1B_ and 5-HT_2C_.

5-HT_1B_ and 5-HT_2C_ receptors have been shown in several studies to exert an inhibitory effect on ejaculation [31,32,33]. This has primarily been established by studying the effect of administration of receptor agonists in rat models. In particular, Hillegaart and Ahlenius established that subcutaneous administration of a 5-HT_1B_ receptor agonist, anpirtoline, was found to impair ejaculation in rats [31]. Moreover, Foreman et al. demonstrated that systemic administration of DOI, a 5-HT_2A/2C_ receptor agonist also suppressed ejaculation in rats [32]. It is important to note that both of these receptor subtypes are distributed in the lumbosacral spinal cord, sacral parasympathetic nucleus and the hypothalamus, as well as other spinal and supraspinal areas. It has not yet been established whether inhibitory action predominantly occurs at a supraspinal or spinal level, and further research is necessary to determine this [34].

5-HT_1A_ receptors are found in high densities in the raphé nuclei where they are thought to primarily exert their effect on ejaculation. Unlike the previously discussed receptor subtypes, stimulation of the 5-HT_1A_ receptor has been found to primarily exert a pro-ejaculatory effect. It has been demonstrated that administration of 8-OH-DPAT, a 5-HT_1A_ agonist, decreased both the frequency of intromissions, as well as decreased ejaculatory latency time in a rat population [31]. It has also been found that 5-HT_1A_ receptor agonists inhibit penile erections [35]. In addition to being found in high densities in the raphe nuclei, these receptors are also present in spinal centres, including within the dorsal horn, sacral parasympathetic nucleus and in the dorsal grey matter commissure in the lumbosacral spine. However, more research into the functional role of these spinal sites is necessary to establish their effect.

### 5.2. Selective Serotonin Reuptake Inhibitors

This inhibitory effect has been harnessed for the development of pharmacological treatments for PE. The aim of pharmacological treatment is to increase synaptic levels of serotonin. Selective serotonin re-uptake inhibitors (SSRIs), have been prescribed for the treatment of psychiatric disorders including depression and anxiety. SSRIs bind to serotonin transporter (5-HTT) and prevent the re-uptake of serotonin into the presynaptic axon terminal. By doing this, SSRIs increase synaptic levels of serotonin, leading to increased post-synaptic nerve transmission. A well-documented side effect of this treatment is sexual dysfunction, including delayed ejaculation [36]. On this basis, SSRIs have been successfully used for the treatment of premature ejaculation. This form of pharmacotherapy is primarily indicated for patients with lifelong PE.

Dapoxetine is the first compound specifically designed for the management and treatment of premature ejaculation. It is a fast-acting SSRI with a short half-life approved in many countries around the world including Australia, New Zealand and many countries in the European Union. It is administrated orally as needed (prn) 1–3 h before intercourse, indicating it may be effective as an ‘on-demand’ treatment for PE. The drug also shows dose-proportional pharmacokinetics, as shown in clinical trials using 30 mg and 60 mg doses [37]. In phase II and phase III clinical trials, dapoxetine has shown to be efficacious in treating all components of premature ejaculation in patients with lifelong PE [37,38,39,40,41]. This includes significantly increasing IELT, increasing perceived control over ejaculation, as well as decreasing the negative personal consequences, such as distress, surrounding PE. Subsequent clinical study evidence of dapoxetine has reinforced results of the clinical trials [42,43,44].

In addition to blocking synaptic serotonin reuptake, radioligand binding studies in human cells have demonstrated that dapoxetine also binds to norepinephrine (noradrenaline) (NE) and dopamine (DA) reuptake transporters [45]. This may indicate peripheral actions of dapoxetine, however the effect of dopaminergic and norepinephrine (noradrenaline) reuptake inhibition is reported to be minimal [46].

### 5.3. Other Proposed Pharmacological Management Options

Other pharmacological treatments for PE have been identified but are not currently approved as effective treatments for PE. Tricyclic antidepressants (TCAs) have been shown to be effective in the treatment of PE, however their use is limited due to a significant side effect profile, including anti-cholinergic effects such as nausea, dry mouth and blurred vision, as well as cardiotoxicity and erectile dysfunction. Clomipramine is the primary agent used as off-label treatment of PE in patients, and has shown to be effective in increasing IELT [47]. Tramadol has also been trialled as a potential treatment for PE. The exact mechanism of action is unknown, however it has been postulated that Tramadol may act as a mu-opioid agonist, a 5-HT_2C_ receptor antagonist, as well as a serotonin and norepinephrine (noradrenaline) modulator [48]. However, as the research base is limited, more studies are necessary to determine the agent’s safety and efficacy for use in this population of patients. Phosphodiesterase-5 inhibitors have also been trialled in order to improve the patient’s sense of control over ejaculation, however, indication as a pharmacological strategy of PE management remains controversial [49].

### 5.4. Psychological and Psychosexual Behavioural Therapy

Psychological therapy is another treatment option which may benefit a patient with PE. This treatment option may best be indicated for patients with subjective or natural variable PE, or acquired PE in which interpersonal or psychological causes may be an underlying factor contributing to PE [50]. In this form of management, deeper psychological and interpersonal causes surrounding PE can be elicited and explored with the assistance of the clinician. Psychological therapy may be particularly efficacious in reducing the distress caused by PE. However, the evidence supporting psychological approaches for PE management is inconsistent and lacking in long-term follow-up [51].

Psychosexual behavioural methods have also been used as additional treatment options for PE. This treatment option focuses on the education of patients on techniques to delay ejaculation. These methods include the ‘start–stop’ program, developed by Semans [52]. In this technique, which may be referred to as ‘edging’, sexual intercourse begins and progresses to a point near orgasm/ejaculation. At this point, all sexual stimulation is stopped until the feeling passes, and sexual intercourse may resume. Another described technique is the ‘squeeze’ or ‘stop–squeeze’ technique, first proposed by Masters and Johnson [53]. In this technique, intercourse progresses to the point of near orgasm/ejaculation. At this point, the penis is removed from the vagina and the glans of the penis is squeezed. Another common technique often used by younger males to delay ejaculation is masturbation before anticipated intercourse. This method can become more difficult to implement as the age-related increase in refractory time, the recovery phase after ejaculation during which it is physiologically impossible to have additional orgasms, progresses.

These treatment strategies can be partially effective in the management of PE. However, behavioural or psychological therapy alone is usually not adequate to entirely manage and treat PE. Despite showing high success rate of up to 65% for short-term management of PE, the long term success seems to be relatively poor, effectively managing only 25% of patients [54]. It should also be noted that the reported success rates have been difficult to duplicate and verify in subsequent studies [55]. There are also several limitations to psychosexual behavioural therapy [56]. Firstly, the efficacy of the treatment is uncertain, and may not be effective in managing certain patients. Moreover, the therapy requires time and repeated practice to become effective. This time requirement, as well as the need for multiple sessions with an expert may be time consuming and costly for the patient, which may discourage the use of this treatment option. Finally, the techniques often require a partner to assist in the management process, which may present an issue if the partner is unwilling or unable to invest the time.

## 6. Patient Management

**Figure 3:**
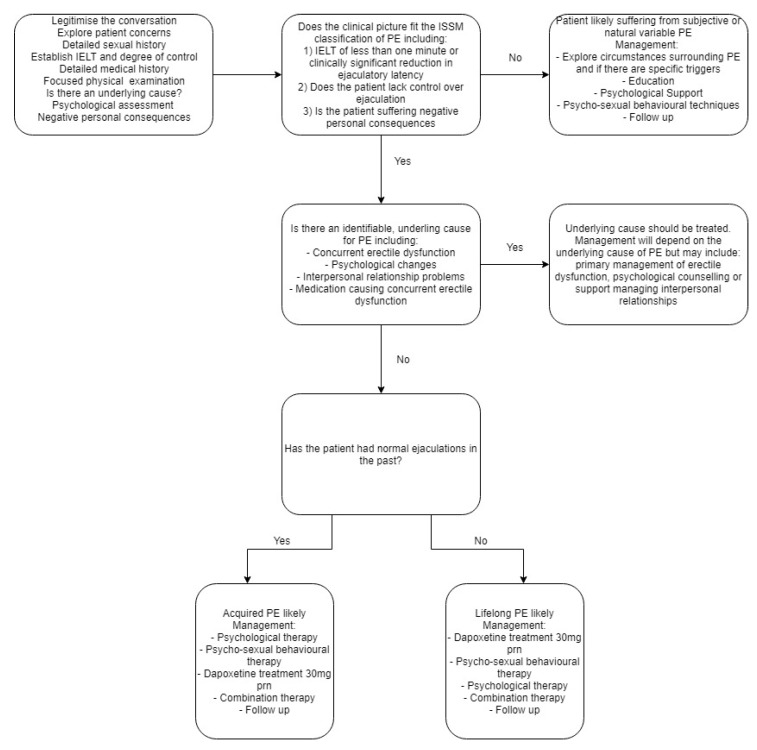
Suggested management algorithm for patients presenting with suspected PE.

The management of a patient presenting with PE will depend on the cause and classification of PE. (see Figure 3). It is therefore important for the clinician to firstly determine if there is an underlying cause for the PE. Common underlying causes include concurrent erectile dysfunction, sexual performance anxiety and interpersonal relationship problems [13]. The underlying cause of premature ejaculation should be managed first, followed by secondary treatment of PE if the symptoms do not remediate after treating the primary causes. It is also important during the management stage to set treatment goals with each patient and partner if appropriate, in order to better guide therapeutic actions.

### Combined Therapy for the Treatment of Premature Ejaculation

A strict division between pharmacological and non-pharmacological treatment for PE may not be the most effective way of treating patients with PE. As the effects of PE on a patient and their partner/s is multifactorial, exclusive treatment may fail to address certain patient needs. In particular, sole pharmacological treatment may be unable to address significant emotional distress surrounding PE, interpersonal relationships, and unrealistic expectations from patients. Psychological and psychosexual behavioural strategies may similarly fall short in adequately extending IELT in patients or increasing the patient’s perceived control over ejaculation. A suggested solution to providing holistic patient care is combination or integrated therapy. A study by Cormio et al. found that combination therapy of Dapoxetine and psychosexual behavioural treatment was more effective than pharmacological treatment alone in improving IELT in patients with lifelong PE [57]. Patients receiving 30 mg Dapoxetine were compared with patients receiving 30 mg of Dapoxetine combined with psychological intervention. It was found that pharmacological treatment alone increased mean IELT from a baseline of 85.0 s to 160.0 s over 24 weeks, while combination therapy increased IELT from a baseline of 92.0 s to 370.7 s. Although this study did not use sense of ejaculatory control and patient distress associated with PE as an outcome measure, the study does indicate the promise of combination therapy. A combined treatment of pharmacological, and non-pharmacological management may be superior to pharmacological treatment alone in providing patients with holistic and effective care.

## 7. Conclusions

PE is a highly prevalent male sexual disorder that is largely an unmet therapeutic need. Patients with PE are characterised as having low IELT, lacking control over ejaculation and suffer from negative personal consequences as a result of the condition. PE can affect sexual and psychological health, as well as interpersonal relationships. As the effects of PE are multifactorial, a holistic patient management plan composed of pharmacological management, psychological support and psychosexual behavioural therapy is likely to produce the best outcomes for patients.
